# The Clinical Importance of the Mercury Problem in Artisanal Small-Scale Gold Mining

**DOI:** 10.3389/fpubh.2019.00131

**Published:** 2019-05-29

**Authors:** Justice Afrifa, Yeboah Kwaku Opoku, Eric Ofori Gyamerah, George Ashiagbor, Rosemary Doe Sorkpor

**Affiliations:** ^1^Department of Medical Laboratory Science, School of Allied Health Sciences, University of Cape Coast, Cape Coast, Ghana; ^2^Scientific Research Center, Second Affiliated Hospital of Harbin Medical University, Harbin, China; ^3^Department of Biomedical Science, School of Allied Health Sciences, University of Cape Coast, Cape Coast, Ghana; ^4^Biopharmaceutical Laboratory, College of Life Sciences, Northeast Agricultural University, Harbin, China; ^5^Department of Biochemistry, School of Biological Sciences, University of Cape Coast, Cape Coast, Ghana; ^6^Department of Wildlife and Range Management, Kwame Nkrumah University of Science and Technology, Kumasi, Ghana; ^7^Food and Drugs Authority, Cape Coast, Ghana

**Keywords:** mercury toxicity, artisanal small-scale mining, organ dysfunction, gold, PPE compliance

## Abstract

Artisanal small-scale mining is widely operated in various countries serving as a livelihood to many rural communities. However, it is a significant source of environmental mercury contamination which affects human health. Amalgamation and amalgam smelting, two significant steps in the artisanal small-scale mining operations generate lots of mercury vapors, leading to chronic exposure among miners. Thus, this article seeks to provide a topical review of recent findings on organ damage and metabolic disorders among mercury-exposed artisanal small-scale miners with emphasis on the contributing factors such as personal protective equipment usage and artisanal small-scale gold mining-specific occupational activities. Also, insights into the effect of mercury intoxication and mechanisms of action on organ and metabolic systems among exposed individuals are provided.

## Introduction

Artisanal or small-scale gold mining (ASGM) is usually characterized by the use of rudimentary or basic tools operating at a limited level of production with reduced capital investment. According to the International Labor Organization, ASGM operations are labor intensive, employing low, and basic level mechanization (1). In most instances, poverty is an influential primary drive in ASGM activities thus providing a source of livelihood for many rural communities (2, 3). The economic importance of ASGM cannot be overemphasized, hence the extensive coverage of its operation spanning over 55 countries across Asia, South America, and Africa (4). It is reported that direct or indirect dependency on ASGM for livelihood exceeds 100 million persons globally (5). Besides these essential microeconomic effects, other adverse environmental and health impacts are associated with ASGM. Numerous studies have reported on the contributions of ASGM to land degradation (6), and water pollution (7–9), as well as associated social problems (10). The incidence of increased social aggression and violence between resident miners and new settlers or larger mining companies have been reported (11). Irrespective of these issues, it appears that strict regulations needed to streamline the activities and operations of ASGM are not adequate and sometimes fails to protect the natural ecosystems of the affected areas (9).

Of particular concern in AGSM is what is described as the “mercury problem” and its clinical implications on human health. As much as ASGM provides a livelihood to many rural communities, it is also responsible for the world's fastest emerging source of mercury contamination (2, 3). Usually, the type of mining procedure employed by miners of various precious minerals on a small scale is mainly dependent on the characteristics of the mining site and the type of mineral involved. In ASGM, gold amalgamation with mercury has been a critical component in the extraction process (12). Global estimation of the release of airborne elemental mercury attributed to inappropriate mining practices is about 400 tons (13). The WHO reports implicated the inhalation of a vaporized form of elemental mercury during amalgam smelting as the main route of exposure (14). However, exposure through other routes including oral and dermal is also possible (15). Mercury toxicity is known to be insidious, hence the transition from acute to chronic exposure may be silent. Trends in current studies point to increasing disruption of organ and metabolic functions among artisanal small-scale miners (ASGMs) as a result of mercury exposure. It is therefore vital that various clinical implications of mercury exposure and the associated damaging effects among ASGMs received the necessary attention. Thus, this article seeks to provide a literature review of the recent findings on the contribution of mercury exposure to organ and metabolic disorders among ASGMs with emphasis on contributing factors such as personal protective equipment (PPE) usage and ASGM-specific occupational activities. In addition, we provide insight into the effect of mercury intoxication and mechanisms of action on organ and metabolic systems among exposed individuals. Relevant documents pertaining to the topic under discussion were extracted from Web of Science, Pubmed, and Google scholar. However, relevant studies that assess specific clinical biomarkers among ASGMs are shown in Table 1.

**Table 1:** Health implications of mercury exposure to ASGMs.

**Clinical outcome**	**Effects**	**References**	**Year**	**Country**
Neurologic effects	Increased mercury levels in urine and hair	Bose-O'Reilly et al. (16)	2016	Indonesia
		Peplow and Augustine (17)	2014	Surinam
		Dolbec et al. (18)	2000	Brazil
Renal abnormalities	Reduced eGFR, elevated urine protein	Afrifa et al. (19)	2017	Ghana
	Increase in proteinuria	Ekawanti and Krisnayanti. (20)	2015	Indonesia
	Increased proteinuria	Franko et al. (21)	2005	Nigeria
Thyroid risks	Reduction in total triiodothyronine, free triiodothyronine, and free thyroxine	Tayrab (22)	2018	Sudan
Haemato-pathological risk	Reduced hemoglobin and hematocrit levels	Ekawanti and Krisnayanti (20)	2015	Indonesia
	Associated with anemia in children	Weinhouse et al. (23)	2017	Peruvian Amazon, Peru
DNA oxidative stress	Positive correlation of LINE1 methylation with 8-hydroxy-2-deoxyguanosine(8-297 OHdG) and Hg levels	Narváez et al. (24)	2017	Colombia
	Alterations in membrane permeability, changes in macromolecular structure	Flora et al. (25)	2008	India
Genotoxic damage	Micronuclei in mouth epithelial cells, nucleoplasmic bridges, gemmation, and binucleation	Rosales-Rimache et al. (26)	2013	
Immunological response	High ANA and ANoA, increased titers of ANA and ANoA, higher levels of proinflammatory cytokines IL-1β, TNF-α, and IFN-γ	Gardner et al. (27)	2010	Brazil
	Increased pro-inflammatory, anti-inflammatory cytokines, and IL-17	Nyland et al. (28)	2011	Brazil

## Mercury Speciation, Toxicity, and the “Mercury Problem” in ASGM

### Mercury Speciation and Toxicity

Mercury is an environmental toxin which is widely distributed and has been at the center of recent public health discussions due to the severity of its impact on human health systems. Physiologically, mercury seems to have no known beneficial health effect, even though an average man weighing 70 kg is reported to accumulate about 13 mg of it (29). Beside the immunotoxic effect, the level of toxicity is such that it affects all manner of persons and even low exposure levels are known to cause toxicities to the cardiovascular, reproductive, developmental, renal, and central nervous systems (CNS). Mercury poisoning and severity depends on the chemical state of the mercury which determines the route of exposure. The environmental distributions are in three forms(elemental, organic, and inorganic), and each is characterized by specific chemical reactions, solubility, and toxicity (30). Elemental mercury, also known as quicksilver, is liquid at room temperature and is poorly absorbed in the gastrointestinal tract (GIT) rendering it less toxic in this form and hence quickly eliminated through fecal matter upon ingestion. However, when volatilized into a vapor, it is well-absorbed through the lungs (31). Mercury vapor, when inhaled, could quickly enter the circulatory system via the pulmonary alveolar membranes and invade the CNS, blood cells, and the kidneys where it can be partially converted to HgCl_2_ and retained for years (32). Elemental mercury exposure is monitored through the measurement of urine mercury. Chronic intoxication could exhibit a myriad of symptoms ranging from polyuria, and proteinuria to nephritic syndromes in severe cases accompanied with hematuria and anuria (33).

Organic mercury in the form of methyl, ethyl or phenylmercury is usually formed through the biomethylation of mercuric compounds by aquatic microorganisms and eventually released into the environment. Among the various forms, methylmercury seems to be the most toxic with extensive bioaccumulation along the sequential order through the aquatic food chain. Substantial accumulation is observed in highest order fish predators and other fish consuming marine mammals. According to the committee on environmental studies and toxicology of the national research council in the year 2000, consumption of fish contaminated with mercury had led to a lot of public health concerns such as neurotoxicity (34). Unlike elemental mercury, up to about 95% of methylmercury is absorbed by the GIT into the red blood cells and the brain through a covalent bond with glutathione and cysteine protein groups (35) The level of blood mercury is an indicator of total mercury, which is made up of both elemental and methylmercury. However, methylmercury usually constitutes a significantly higher percentage of total blood mercury. Nonetheless, there is evidence to show that both inorganic and organic forms of mercury could be predicted from total blood mercury (36). For non-fish-eating people, the International Union of Pure and Applied Chemistry (IUPAC), in collaboration with the International Commission on Occupational Health (ICOH), set the maximum limit of blood mercury concentration at 2 ug/l (37). People intoxicated with methylmercury could exhibit symptoms such as ataxia, peripheral neuropathy, choreoathetosis, visual loss, confusion, and coma (38).

Inorganic mercury, usually referred to as mercury salts, are useful for several products including medicines, explosive detonators, and mercuric fulminates (39). However, it could have very deleterious effects on human health upon exposure. There is evidence to show that, elemental mercury could be converted into other forms or combine with Sulfur compounds *in vivo* (40, 41). Also, compounds containing both organic and elemental mercury could be oxidized to the inorganic forms resulting in the production of hydroxyl radicals (42). Mercurial is excreted through the fecal matter hence their negligible excretion from the urine leading to reduced levels (about 10%) of total excretion (30). However, in the state of chronic exposure, there is an increase in excretion levels (43). The ability of inorganic mercury compounds to bond with sulfhydryl (-SH) groups and the degree of its solubility determines its degree of toxicity. Mercury salts primarily target the kidneys leading to acute tubular necrosis, nephrotic syndrome, and general renal injuries. Chronic exposure could also cause permanent damage to the CNS.

### Mercury Use and Exposure Among ASGM

ASGM is a long-standing operation with the main focus to extract gold from sediments or rocks. However, operators usually work on secondary or tertiary alluvial ores located in river sediments. This is achieved by panning, dredging, or hosting sediments down river banks or open pits by the use of high-pressure pumps. Briefly, the process of ASGM involves a series of steps including extraction of ore from the soil, crushing of the ore using rudimentary tools, milling of the ore into fine powder, concentration and washing in sluices and pans, panning, amalgamation to capture the gold and lastly burning the amalgam to recover the gold. For the purpose and objectives of this review, we focus on the processes that employ the use of mercury which may lead to occupational exposure.

In most ASGM, mercury is used in the amalgamation process, and the amount of environmental emission depends on the method employed. In whole ore amalgamation, there is enormous environmental emission of mercury as opposed to the gravity amalgamation method. In either method, mercury is emitted into the environment from the disposal of mercury-laced tailings and process water into water bodies and underground vicinities coupled with atmospheric emission of mercury vapor from the smelting process (9). It is estimated that for every gram of gold produced by amalgamation, 1 or 2 grams of metallic mercury is lost (44).

Occupational and general population exposure to mercury can be direct and indirect. Direct exposure is chiefly attributed to the presence of elemental mercury (Hg) vapors in ambient air. Notwithstanding, the inhalation of mercury vapor in workplace atmospheres is the main route of occupational exposure which is often severe and acute with up to 80% absorption by the lungs during high exposure (45). The major organs damaged are mostly the kidneys and lungs, but there can also be damage to the nervous system. According to the WHO, indirect exposure, however, occurs as a result of the presence of elemental mercury vapor (Hg) contaminating surrounding surfaces, getting into water bodies, fishes, foodstuffs, etc. (46). The primary and major sources of all non-occupational exposures are through dietary intake of contaminated fishes and seafood (47). Heating of mercury amalgam also unleashes mercury vapors into the atmosphere which are deposited on walls, clothes and other surrounding surfaces leading to environmental contamination. Contact with any of these contaminated objects even after burning the amalgam could lead to inhalation of the fumes. The effect of mercury exposure is not limited to the ASGMs but also the surrounding communities, as the vapors are blown by the wind to nearby surroundings.

## Specific Occupational Activity and PPE Non-Compliance Among Mercury-Exposed ASGMs

In a high mercury polluted environment, the use of PPE should be strictly adhered to mitigate the personal exposure level. However, reports among ASGMs reveal an insignificant to a non-existent PPE compliance. For instance, among ASGMs in Ghana, Afrifa et al. found that about 86.55% of their study participants exhibited absolute non-compliance for appropriate use of protective clothing or equipment. Specifically, 89.8% non-compliance in the use of nose mask for protection against vaporous mercury was reported (19). These findings are similar to those of earlier studies which reported limited use of PPE among ASGMs (3). (48) confirms that, among a group of Tanzanian ASGM population, miners and millers were directly exposed to mercury during both amalgamations and burning without retorts (a device that collects mercury during amalgam burning) (48). This trend seems to be widely spread across the ASGM communities around the world as Rojas et al. (49) also found about 55.5% of absolute PPE non-compliance among gold workers near El Callao, Venezuela. The high non-compliance with PPE use has been attributed to the low level of formal education as well as lack of training in mercury toxicity and management processes among ASGMs. Interestingly, there seems to be a reluctance among ASGMs to employ the use of a retort which could provide a medium to reduce exposure to vaporous mercury. Even though it is less expensive, easy to use, and globally recommended, non-compliances have been reported in Ghana (3), Mozambique, Ecuador, and Guyana (50).

Among ASGMs the level of mercury exposure and subsequent intoxication is associated with the specific type of job and at which stage of the mining process an individual is engaged in. Again, occupational mercury exposure has been associated with factors such as the type and nature of mercury, the number of years of exposure as well as the physical state of the mercury (51). In its natural state and at room temperature, liquefied mercury, also known as quicksilver, is less toxic when ingested. This is due to the inability of the gastrointestinal tract to readily absorb it, leading to complete excretion in the stool. However, there is conclusive evidence to show that activities that could lead to the generation of vaporous mercury such as the heating and burning of mercury are associated with increased mercury exposure and subsequent intoxication. This assertion is supported by findings from different studies that reported increased mercury exposure among ASGMs that are involved in gold smelting and amalgamation (19, 20). Again, the risk of increased mercury exposure and intoxication has been associated with the duration of work as an artisanal gold miner. Basically, the longer the working period, the higher the risk of exposure. Franko et al. (21) found that among ASGMs at the Idrija mines, the duration for the initiation of clinical symptoms was around 15 years. These findings confirm the insidious onset of clinical symptoms that could result in chronic effects with prolonged exposure to mercury vapor. The negative effect of mercury exposure is buttressed by the low ACGIH TLV-TWA exposure limit of 0.025 mg/m^3^ for elemental and inorganic mercury (52) and a BEI of 20 μg Hg/g creatinine for urine mercury (53).

## Health Implications of Mercury Exposure to ASGMs

The use of mercury goes back to ancient times with its widespread medicinal applications in the treatment of conditions such as skin diseases and syphilis. However, with the accompanying side effects and recorded deaths, the harmful effects of mercury exposure on health were evident with much research after the 20th century (54). It was then established that although mercury is widely distributed in the environment, it is non-essential and toxic to the human body (55). ASGMs who solely rely on the use of mercury serves as the major source of global mercury pollution (56). Mostly without any PPEs, the isolation of the amalgam is done manually followed by heating with a torch or over a stove to distill the mercury and isolate the gold. This, therefore, presents myriads of health challenges to ASGMs.

### Neurotoxic Risk of Mercury-Exposed ASGMs

The adverse health implications of mercury and methylmercury are not immediately noticeable but manifested over time (57), up to about 5 to 10 years after exposure (16). Mercury toxicity has been reported to affect various organs and metabolic functions. The most commonly reported complications of mercury intoxication among workers engaged in ASGM are neurologic effects including tremor, ataxia, memory problems, and vision disorders (57). Among people living in Cisitu, a small-scale gold mining village in Indonesia, Bose-O'Reilly et al. reported severe neurological symptoms and elevated levels of mercury in urine and hair. This was reported to have resulted from possible exposure to inorganic mercury in the air, and the consumption of mercury-contaminated fish and rice (16). Elsewhere among the indigenous Wayana community of Puleowime-Surinam, neurological abnormalities were observed among the mercury-exposed population. The researchers through a battery of neurological tests reported neurotoxic effects which were consistent with methylmercury exposure (17). These findings were consistent with those of previous studies which concluded that neurobehavioral manifestations consisting of subtle neurotoxic effects on motor functions among people living in gold mining areas of the Brazilian Amazon were associated with low-level methylmercury exposure (18). Clinically, several plausible reasons could contribute to the neurodegenerative effect of mercury exposure among these miners. These may include, but are not limited to, the ability of mercury [especially methylmercury (MeHg)] to increase the level of reactive oxygen species. This is evident from the fact that the treatment of mice with methylmercury led to an upsurge in lipid peroxides in the brain, coupled with the inhibition of antioxidant enzymes (58). Methylmercury impedes glutathione reductase (59) and binds directly to selenocysteine residue in glutathione peroxidase (GPx), leading to an increase in hydrogen peroxide levels (60). Again, methylmercury is reportedly involved in the inhibition of thioredoxin and thioredoxin reductase preventing the elimination of ROS (61). Lastly, it is possible for MeHg to impede the structuring of microtubules which are essential for CNS development (62–64). On the other hand, inorganic mercury has been implicated in neuronal hyperpolarization by increasing the permeability of chloride channels of GABA_A_ receptors in the dorsal root ganglion (65).

### Hepato-Renal Risk of Mercury-Exposed ASGMs

Besides the central nervous system, the kidneys are easily targeted for mercury toxicity with high accumulation particularly in the areas of the proximal tubules (66). However, the extent of damage and renal dysregulation have been shown to be a function of the duration of exposure, state of the mercury, and the extent of mercury exposure, as well as the exposure route (67). Among small-scale gold miners, renal abnormalities have been expressed in the form of elevated serum creatinine, reduced eGFR and increased proteinuria (68). This was in line with a recent study which reported a significant association of mercury exposure with reduced eGFR, and elevated urine protein among ASGMs in Ghana (19). Similarly, a significant increase in proteinuria has been reported among mine workers in Idrija, Nigeria (21) and Sekotong, West Lambork-Indonesia (20). In contrast, others reported no association of low to moderate mercury levels with renal function biomarkers such as creatinine, albumin, and excretion of β-2 macroglobulin except a significantly reduced eGFR among artisanal gold miners who are exposed to mercury vapor (67). Again, a study that assessed exposure to airborne mercury during gold mining in Venezuela found 7.8% of the participants presenting with a detectable level of urinary N-acetyl-ß-D-glycosaminidase (NAG). They also reported a significant correlation between urine NAG and urine mercury levels among the study participants (69). The advantage of NAG as a renal biomarker is its ability to detect preclinical, non-specific cellular disruption to the proximal tubules of the kidneys. This confirms the insidious nature of mercury toxicity, hence the need for precautionary measures to be adopted by small-scale miners. Within the kidney, the most vulnerable section susceptible to mercury toxicity is the pars recta of the proximal tubules. Experimental studies in animal models have revealed immunologically mediated glomerulonephritis after mercury exposure (70), emphasizing the link between mercury exposure and glomerulonephritis, chronic renal disease, acute tubular necrosis, and nephrotic syndrome (71–73). Studies have also reported various kidney injuries such as tubular dysfunction, increased proteinuria as well as glumerulonecrosis among mercury-exposed workers (74).

### Thyroid and Haemato-Pathological Risk of Mercury-Exposed ASGMs

Thyroid hormones critically influence brain development through its involvement in neurogenesis and synaptogenesis with classical function in cellular differentiation, metabolic regulation, growth, reproduction, and mediation of cellular response to stimuli. Among small-scale miners, few studies (22, 75) have assessed the possible relationship between thyroid function and mercury exposure. First, Tayrab reported a significant increase in thyroid stimulating hormone and total thyroxine with a consequent reduction in total triiodothyronine (T3), free triiodothyronine (FT3), and free thyroxine (FT4) irrespective of age and weight among traditional gold miners working in Abuhamed-Sudan (22). Others have reported an association between elevated blood mercury levels and variations in thyroid hormone levels among artisanal small-scale miners in Ghana. The above study also found that increasing blood mercury levels corresponded with a decrease in both FT4 and FT3. However, they reported a normal mean FT3 and FT4 among the miners and, thus, immediate clinical complication was not inferred (75). Again, evidence of T3 reduction in response to mercury exposure has been reported in several studies (76, 77). Mercury can interfere with hormone receptor action, thus contributing to the inhibition of some specific enzymes in the hormonal synthetic pathways with the possibility of endocrine function impairment (78). Findings from the above studies point to a decreased level of T3 among the mercury-exposed groups compared to the unexposed controls. It is possible for mercury to inhibit 5′- thyroxine deiodinase type II, which is critical in the conversion of T4 to T3, by primarily targeting the liver (79). Also, numerous studies (80–82) have provided evidence of deiodinase activity inhibition mediated by mercury exposure in adult rat liver. After inhalation, mercury vapor is rapidly dispersed into the blood with subsequent depositions in organs including brain, kidney, and placenta (21, 55). Also with relative ease, inhaled mercury vapor enters the circulatory system and invade primary red blood cells by crossing the pulmonary alveolar membranes (32). Ekwanti et al. reported a significantly reduced hemoglobin and hematocrit levels among mercury-exposed small-scale miners compared to non-miners and also found a significant correlation between the smoking habits of miners and hemoglobin concentration. However, the seemly abnormal HB levels of the mercury-exposed miners were restored when corrected for smoking (20). Elsewhere, Brandt al. reported a reduction in hematocrit and hemoglobin levels in mercury-exposed mice (83). In a recent study conducted in the Peruvian Amazon, the level of hair methylmercury was associated with anemia in children living close to an ASGM community (23). Further, it has been established that the ability of mercury to compete with iron for binding to hemoglobin results in impaired hemoglobin formation (84).

### DNA Oxidative Stress and Genotoxic Risk Among Mercury-Exposed ASGMs

Apart from the regular organ function biomarkers such as thyroid hormones, liver enzymes, and regular renal function markers, other markers have been employed to assess the level of ASGM associated mercury toxicity. Mercury is a known precursor to oxidative stress and mitochondrial functional abnormality (85), and this may result in an alteration in calcium homeostasis that leads to lipid peroxidation. The ability of mercury to also act as a catalyst for Fenton-type reaction promotes the levels of radical oxygen species thus increasing the oxidative stress level (86). In a study that assessed oxidative stress and repetitive element methylation changes in artisanal gold miners occupationally exposed to mercury in Colombia (La Mojana), Narvaez et al., reported a significant association of Long Interspersed Nuclear Element 1 (LINE1) and Alu(Yb8) DNA methylation with gold miners as compared to the control group. Additionally, LINE1 methylation was positively correlated with 8-hydroxy-2-deoxyguanosine(8-OHdG) and Hg levels. (24) These findings emphasize the negative effect of Hg on oxidative stress and DNA methylation in gold miners. Various studies (25, 87, 88) have reported alterations in membrane permeability as well as changes in macromolecular structure due to the high affinity of cellular mercury for sulfhydryl and thiol groups leading to DNA damage. This has been demonstrated by the significant increase in 8-OHdG in the urine of mercury-exposed workers in China (89) similar to that observed by Narvaez and co.

In another study, mercury exposure related to genotoxic damage among small-scale mining workers was reported. Rimache et al. found that as much as 15% of their study population presented micronuclei in mouth epithelial cells. Their results also indicated the presence of other chemical risk genotoxic indicators including nucleoplasmic bridges, gemmation, and binucleation (26).

### Immunological Response to Mercury Exposure Among ASGMs

Mercury is an immunotoxin which has been shown to induce an autoimmune response in animal models (90). The immunotoxicity of mercury is characterized by lymphoproliferation, elevated immunoglobulin (IgG and IgE) levels, as well as increased circulating levels of autoantibodies such as anti-nuclear autoantibodies (ANA) and anti-nucleolar autoantibodies, (ANoA) (91). Recent studies among mercury-exposed small-scale miners reveal a higher prevalence of detectable ANA and ANoA and increased titers of ANA and ANoA among mercury-exposed gold miners compared to diamond and emerald miners who are not at risk of occupational mercury exposure. The researchers also identified higher levels of proinflammatory cytokines IL-1β, TNF-α, and IFN-γ in the serum of the mercury-exposed gold miners compared to diamond and emerald miners (27). In line with the above study, Nylander and co also reported an association between elevated blood and plasma mercury with ANA but not with ANoA. However, they found that among persons living in the Lower Tapajós River Basin in the Brazilian Amazon with a history of mercury use in small-scale gold mining, pro-inflammatory, anti-inflammatory cytokines, and IL-17 were increased with MeHg exposure (28). The above studies provide evidence and confirmation of the immunomodulatory effect of mercury exposure among the affected population.

### Signs and Symptoms of Mercury Intoxication Among Artisanal Small-Scale Miners

The signs and symptoms of mercury exposure among artisanal small-scale miners have been shown to be a function of the extent and route of exposure (3). The commonly reported symptoms range from hair loss through a persistent headache to skin rashes. Among Ghanaian small-scale miners, itchy eyes, fatigue, skin rashes, red eyes, metallic taste, and complaints of numbness were reported as the predominant complications suggestive of mercury exposure (3, 19). Elsewhere, low-level mercury exposure has been associated with symptomatic central nervous system toxicity in Bornuur and Jargalant soums (92). In Burkina Faso, Tomicic et al. (93) found general complaint of headaches, thoracic pain, dizziness, vision disorder and persistent cough as the most prevalent signs and symptoms among workers who washed ore. They also reported a direct relationship between the task of packaging mercury in small flasks and rhinitis (93). Similarly, fine tremor of the eyelids, fingers, and lips, altered tendon reflexes and dysdiadochokinesis in addition to metallic taste and sleep disturbances were observed among mine workers at Rwamagasa (48).

## Conclusion and the Way Forward

Mercury exposure presents myriad of health disorders ranging from neurotoxicity and hepato-renal dysfunction, to thyroid and haemato-pathological disorders of public health concern. This comes about as mercury interferes with the action of some important biological molecules and also causes oxidative stress, leading to these observed health implications. However, the use of PPEs which can reduce the level of exposure to mercury is very limited among ASGMs, leading to excessive exposure. It is, therefore, vital that these miners are encouraged to adhere to the use of PPEs and other safety devices such as retorts which could reduce the level of vaporous mercury exposure. Most importantly, gold mining procedures devoid of mercury use, such as cyanide leaching and floatation techniques to recover gold, should be encouraged and regulated among these workers. Finally, it seems the level of knowledge on the harmful effects and management of mercury among these miners is low. It is, therefore, important that regular training and workshops are organized for these miners to help increase the level of awareness to the inherent dangers of the use of mercury both on human health and the entire ecosystems.

## Author Contributions

JA conceived and designed the manuscript, JA, YO, EG, GA, and RS wrote, reviewed and edited the manuscript.

### Conflict of Interest Statement

The authors declare that the research was conducted in the absence of any commercial or financial relationships that could be construed as a potential conflict of interest.

## References

[1] Jennings I Report for Discussion at the Tripartite Meeting on Social and Labour Issues in Small-scale Mines. Geneva: International Labour Organization (1999).

[2] Swain EB Jakus PM Rice G Lupi F Maxson PA Pacyna JM. Socioeconomic consequences of mercury use and pollution. Ambio. (2007) 36:45–61. 10.1579/0044-7447(2007)36[45:SCOMUA]2.0.CO;2 17408190

[3] Mensah EK Afari E Wurapa F Sackey S Quainoo A Kenu E. Exposure of small-scale gold miners in prestea to mercury, Ghana, 2012. Pan African Med J. (2016) 25:6171. 10.11604/pamj.supp.2016.25.1.6171 28210374 PMC5292116

[4] Hilson G Garforth C ‘Agricultural poverty'and the expansion of artisanal mining in Sub-Saharan Africa: experiences from Southwest Mali and Southeast Ghana. Populat Res Policy Rev. (2012) 31:435–64. 10.1007/s11113-012-9229-6

[5] Chupezi TJ Ingram V Schure J Impacts of Artisanal Gold and Diamond Mining on Livelihoods and the Environment in the Sangha Tri-National Park Landscape: CIFOR. Bogor (2009).

[6] Arah IK editor. The impact of small-scale gold mining on mining communities in Ghana. African Studies Association of Australasia and the Pacific (AFSAAP). In 37th Annual Conference. Dunedin (2014).

[7] Mujere N Isidro M Impacts of artisanal and small-scale gold mining on water quality in mozambique and zimbabwe. impact of water pollution on human health and environmental sustainability. IGI Global. (2016) 101–19. 10.4018/978-1-4666-9559-7.ch005

[8] Lobo FdL Costa M Novo E Márcia LdM Telmer K Distribution of artisanal and small-scale gold mining in the Tapajós River Basin (Brazilian Amazon) over the past 40 years and relationship with water siltation. Remote Sens. (2016) 8:579 10.3390/rs8070579

[9] Macdonald KF Lund MA Blanchette ML Mccullough CD Regulation of Artisanal Small Scale Gold Mining (ASGM) in Ghana and Indonesia as Currently Implemented Fails to Adequately Protect Aquatic Ecosystems. Xuzhou: Press CUoMaT (2014).

[10] Hoedoafia MA Cheabu BSN Korang V The effects of small scale gold mining on living conditions: a case study of the west gonja district of Ghana. Int J Social Sci Res. (2014) 2:151–64. 10.5296/ijssr.v2i1.4701

[11] Ofosu-Mensah AE Mining as a factor of social conflict in Ghana. J Glob J Hist Cult. (2012) 1: 7–21.

[12] Vieira R Mercury-free gold mining technologies: possibilities for adoption in the Guianas. J Clea Product. (2006) 14:448–54. 10.1016/j.jclepro.2004.09.007

[13] Wuana RA Okieimen FE Heavy metals in contaminated soils: a review of sources, chemistry, risks and best available strategies for remediation. J Isrn Ecol. (2011) 2011:402647 10.5402/2011/402647

[14] WHO U Guidance for Identifying Populations at Risk from Mercury Exposure. Geneva, Switzerland (2008).

[15] Risher JF Amler SN. Mercury exposure: evaluation and intervention: the inappropriate use of chelating agents in the diagnosis and treatment of putative mercury poisoning. J Neurotoxicol. (2005) 26:691–9. 10.1016/j.neuro.2005.05.004 16009427

[16] Bose-O'Reilly S Schierl R Nowak D Siebert U William JF Owi FT. A preliminary study on health effects in villagers exposed to mercury in a small-scale artisanal gold mining area in Indonesia. Environ Res. (2016) 149:274–81. 10.1016/j.envres.2016.04.007 27174690

[17] Peplow D Augustine S. Neurological abnormalities in a mercury exposed population among indigenous Wayana in Southeast Suriname. J Environ Sci. (2014) 16:2415–22. 10.1039/C4EM00268G 25162456

[18] Dolbec J Mergler D Passos C-JS De Morais SS Lebel J. Methylmercury exposure affects motor performance of a riverine population of the Tapajos river, Brazilian Amazon. Int Arch Occupat Environ Health. (2000) 73:195–203. 10.1007/s004200050027 10787135

[19] Afrifa J Essien-Baidoo S Ephraim RKD Nkrumah D Dankyira DO. Reduced egfr, elevated urine protein and low level of personal protective equipment compliance among artisanal small scale gold miners at Bibiani-Ghana: a cross-sectional study. BMC Public Health. (2017) 17:601. 10.1186/s12889-017-4517-z 28655297 PMC5488392

[20] Ekawanti A Krisnayanti BD. Effect of mercury exposure on renal function and hematological parameters among artisanal and small-scale gold miners at Sekotong, West Lombok, Indonesia. J Health Pollut. (2015) 5:25–32. 10.5696/2156-9614-5-9.25 30524774 PMC6221489

[21] Franko A Budihna MV Dodic-Fikfak M. Long-term effects of elemental mercury on renal function in miners of the Idrija Mercury Mine. Ann Occupat Hygi. (2005) 49:521–7. 10.1093/annhyg/mei022 15964879

[22] Tayrab E Thyroid function in sudanese gold miners with chronic mercury exposure. Eur J Pharm Med Res. (2017) 4:177–180.

[23] Weinhouse C Ortiz EJ Berky AJ Bullins P Hare-Grogg J Rogers L. Hair mercury level is associated with anemia and micronutrient status in children living near artisanal and small-scale gold mining in the Peruvian Amazon. Am J Trop Med Hygi. (2017) 97:1886–97. 10.4269/ajtmh.17-0269 29016304 PMC5805048

[24] Narváez DM Groot H Diaz SM Palma RM Muñoz N Cros M-P. Oxidative stress and repetitive element methylation changes in artisanal gold miners occupationally exposed to mercury. Heliyon. (2017) 3:e00400. 10.1016/j.heliyon.2017.e00400 28948237 PMC5602780

[25] Flora S Mittal M Mehta A. Heavy metal induced oxidative stress & its possible reversal by chelation therapy. Indian J Med Res. (2008) 128:501. 19106443

[26] Rosales-Rimache J Elizabeth NM Alarcón J Chávez M Gonzáles M. Genotoxic damage among artisanal and small-scale mining workers exposed to mercury. Revista Peruana de Med Experi Salud Publica. (2013) 30:595–600. 10.17843/rpmesp.2013.304.238 24448935

[27] Gardner RM Nyland JF Silva IA Ventura AM de Souza JM Silbergeld EK. Mercury exposure, serum antinuclear/antinucleolar antibodies, and serum cytokine levels in mining populations in Amazonian Brazil: a cross-sectional study. Environ Res. (2010) 110:345–54. 10.1016/j.envres.2010.02.001 20176347 PMC2873228

[28] Nyland JF Fillion M Barbosa F Jr Shirley DL Chine C Lemire M. Biomarkers of methylmercury exposure immunotoxicity among fish consumers in Amazonian Brazil. Environ Health Perspect. (2011) 119:1733. 10.1289/ehp.1103741 21868305 PMC3261989

[29] Pier SM. The role of heavy metals in human health. J Texas Rep Biol Med. (1975) 33:85–106. 171792

[30] Clarkson TW. The three modern faces of mercury. Environ Health Perspect. (2002) 110:11. 10.1289/ehp.02110s111 11834460 PMC1241144

[31] Hu H. Exposure to metals. Primary Care. (2000) 27:983–96. 10.1016/S0095-4543(05)70185-8 11072295

[32] Clarkson T. The toxicology of mercury. Crit Rev Clin Lab Sci. (1997) 34:369–403. 10.3109/10408369708998098 9288445

[33] Clarkson TW Magos L. The toxicology of mercury and its chemical compounds. Crit Rev Toxicol. (2006) 36:609–62. 10.1080/10408440600845619 16973445

[34] Oken E Bellinger DC. Fish consumption, methylmercury and child neurodevelopment. Curr Opin Pediatr. (2008) 20:178. 10.1097/MOP.0b013e3282f5614c 18332715 PMC2581505

[35] Sarafian T Verity MA. Oxidative mechanisms underlying methyl mercury neurotoxicity. Int J Dev Neurosci. (1991) 9:147–53. 10.1016/0736-5748(91)90005-7 1905456

[36] Halbach S Welzl G. Levels of organic and inorganic mercury in human blood predicted from measurements of total mercury. J Appl Toxicol. (2010) 30:674–9. 10.1002/jat.1540 20981860

[37] Ye B-J Kim B-G Jeon M-J Kim S-Y Kim H-C Jang T-W. Evaluation of mercury exposure level, clinical diagnosis and treatment for mercury intoxication. Annal Occupat Environ Med. (2016) 28:5. 10.1186/s40557-015-0086-8 26807265 PMC4724159

[38] Tekulve K Tormoehlen LM Walsh L Poisoning and Drug-Induced Neurologic Diseases. Swaiman's Pediatric Neurology. 6th edn. Elsevier (2018).

[39] WHO Elemental Mercury and Inorganic Mercury Compounds: Human Health Aspects. Elemental Mercury and Inorganic Mercury Compounds: Human Health Aspects. Geneva: WHO (2003).

[40] Koos BJ Longo LD. Mercury toxicity in the pregnant woman, fetus, and newborn infant: a review. Am J Obstetr Gynecol. (1976) 126:390–409. 10.1016/0002-9378(76)90557-3 786026

[41] Aschner M Lorscheider F Cowan K Conklin DR Vimy M Lash L Metallothionein induction in fetal rat brain and neonatal primary astrocyte cultures by *in utero* exposure to elemental mercury vapor (Hg0) 1. Brain Res. (1997) 778:222–32. 10.1016/S0006-8993(97)01095-0 9462895

[42] Suda I Hirayama K. Degradation of methyl and ethyl mercury into inorganic mercury by hydroxyl radical produced from rat liver microsomes. Arch Toxicol. (1992) 66:398–402. 10.1007/BF02035129 1332650

[43] Dales L Kahn E Wei E Methylmercury poisoning—an assessment of the sportfish hazard in California. California Med. (1971) 114:13. 5544687 PMC1501924

[44] Timmins KJ Artisanal gold mining without mercury pollution. In: UNIDO editor, Organization UNID (2003).

[45] Lorscheider FL Vimy MJ Summers AOJTFJ. Mercury exposure from“ silver” tooth fillings: emerging evidence questions a traditional dental paradigm. FASEB J. (1995) 9:504–8. 10.1096/fasebj.9.7.7737458 7737458

[46] WHO Inorganic Mercury. World Health Organization IPoCS. Geneva, Switzerland (1991).

[47] Richard MP Telmer K Health Issues in Artisanal and Small-Scale Gold Mining: Training for Health Professionals (Version 1.0). Victoria, BC: Artisanal Gold Council (2014). p.1–23

[48] Spiegel SJ. Occupational health, mercury exposure, and environmental justice: learning from experiences in Tanzania. Am J Public Health. (2009) 99:S550–S8. 10.2105/AJPH.2008.148940 19890157 PMC2774175

[49] Rojas M Drake PL Roberts SM. Assessing mercury health effects in gold workers near El Callao, Venezuela. J Occupat Environ Med. (2001) 43:158–65. 10.1097/00043764-200102000-00016 11227634

[50] Kiefer A Drace K Seney C Viega M editors. Challenges Associated with Using Retorts to Limit Mercury Exposure in Artisanal and Small-Scale Gold Mining, Case Studies from Mozambique, Ecuador, and Guyana. In: American Chemical Society Symposium Series. Washington, DC (2015) 10.1021/bk-2015-1210.ch003

[51] Zahir F Rizwi SJ Haq SK Khan RH. Low dose mercury toxicity and human health. Environ Toxicol Pharmacol. (2005) 20:351–60. 10.1016/j.etap.2005.03.007 21783611

[52] ACGIH Threshold limit values (TLVs) for chemical substances and physical agents and biological exposure indices (BEIs). In: 1996: American Conference of Governmental Industrial Hygienists Cincinnati. Cincinnati, OH.

[53] ACGIH Threshold Limit Values (TLVs®) for chemical substances and physical agents and Biological Exposure Indices (BEIs) Appendix B. (2012) 1002.

[54] Bose-O'Reilly S McCarty KM Steckling N Lettmeier B. Mercury exposure and children's health. Curr Prob Pediat Adolescent Health Care. (2010) 40:186–215. 10.1016/j.cppeds.2010.07.002 20816346 PMC3096006

[55] Park J-D Zheng W. Human exposure and health effects of inorganic and elemental mercury. J Prevent Med Public Health. (2012) 45:344. 10.3961/jpmph.2012.45.6.344 23230464 PMC3514464

[56] Esdaile LJ Chalker JM. The mercury problem in artisanal and small-scale gold mining. Chem A Europ J. (2018) 24:6905–16. 10.1002/chem.201704840 29314284 PMC5969110

[57] Gibb H O'Leary KG. Mercury exposure and health impacts among individuals in the artisanal and small-scale gold mining community: a comprehensive review. Environ Health Perspect. (2014) 122:667–72. 10.1289/ehp.1307864 24682486 PMC4080518

[58] Carvalho MC Franco JL Ghizoni H Kobus K Nazari EM Rocha JB. Effects of 2, 3-dimercapto-1-propanesulfonic acid (DMPS) on methylmercury-induced locomotor deficits and cerebellar toxicity in mice. Toxicology. (2007) 239:195–203. 10.1016/j.tox.2007.07.009 17703864

[59] Stringari J Nunes AK Franco JL Bohrer D Garcia SC Dafre AL. Prenatal methylmercury exposure hampers glutathione antioxidant system ontogenesis and causes long-lasting oxidative stress in the mouse brain. Toxicol Appl Pharmacol. (2008) 227:147–54. 10.1016/j.taap.2007.10.010 18023834 PMC2955629

[60] Franco JL Posser T Dunkley PR Dickson PW Mattos JJ Martins R. Methylmercury neurotoxicity is associated with inhibition of the antioxidant enzyme glutathione peroxidase. Free Radical Biol Med. (2009) 47:449–57. 10.1016/j.freeradbiomed.2009.05.013 19450679

[61] Wagner C Sudati JH Nogueira CW Rocha JB. *In vivo* and *in vitro* inhibition of mice thioredoxin reductase by methylmercury. Biometals. (2010) 23:1171–7. 10.1007/s10534-010-9367-4 20717703

[62] Sager P Aschner M Rodier P Persistent, differential alterations in developing cerebellar cortex of male and female mice after methylmercury exposure. J Dev Brain Res. (1984) 12:1–11. 10.1016/0165-3806(84)90170-6 6697246

[63] Ponce RA Kavanagh TJ Mottet NK Whittaker SG Faustman EM. Effects of methyl mercury on the cell cycle of primary rat CNS cells *in vitro*. Toxicol Appl Pharmacol. (1994) 127:83–90. 10.1006/taap.1994.1142 8048057

[64] Kishimoto T Oguri T Tada M. Effect of methylmercury (CH3HgCl) injury on nitric oxide synthase (NOS) activity in cultured human umbilical vascular endothelial cells. Toxicology. (1995) 103:1–7. 10.1016/0300-483X(95)99370-R 8525486

[65] Mottet N. Metabolism of methylmercury in the brain and its toxicological significance. Met Ions Biol Syst. (1997) 34:371–403. 9046576

[66] Nordman H Berlin M Titanium. In Firberg L Nordberg GF Vouk V editors. Handbook on the Toxicology of Metals. New York, NY: Elsevier Science Publishers (1986) 676–718.

[67] Holmes P James K Levy L. Is low-level environmental mercury exposure of concern to human health? Sci Total Environ. (2009) 408:171–82. 10.1016/j.scitotenv.2009.09.043 19850321

[68] Kobal AB Flisar Ž Miklavčič V Dizdarevič T Sešek-Briški A. Renal function in miners intermittently exposed to elemental mercury vapour. Arhiv za Higijenu Rada i Toksikol. (2001) 51:369–80. 11276964

[69] Drake PL Rojas M Reh CM Mueller CA Jenkins FM. Occupational exposure to airborne mercury during gold mining operations near El Callao, Venezuela. Int Arch Occupat Environ Health. (2001) 74:206–12. 10.1007/s004200000206 11355295

[70] Nordman H Berlin M In: Friberg L Seiler G Nordberg H Vouk V editors. Handbook on the Toxiclogy of Metals. Amsterdam: Elseivier (1986). p 594.

[71] Tchounwou PB Ayensu WK Ninashvili N Sutton D Review: environmental exposure to mercury and its toxicopathologic implications for public health. Environ Toxicol. (2003) 18:149–75. 10.1002/tox.10116 12740802

[72] Li S-J Zhang S-H Chen H-P Zeng C-H Zheng C-X Li L-S. Mercury-induced membranous nephropathy: clinical and pathological features. Clin J Am Soc Nephrol. (2010) 5:439–44. 10.2215/CJN.07571009 20089494 PMC2827581

[73] Oliveira D Foster G Savill J Syme P Taylor A. Membranous nephropathy caused by mercury-containing skin lightening cream. Postgraduate Med J. (1987) 63:303–4. 10.1136/pgmj.63.738.303 3684841 PMC2428137

[74] Miller S Pallan S Gangji AS Lukic D Clase CM. Mercury-associated nephrotic syndrome: a case report and systematic review of the literature. Am J Kidney Dis. (2013) 62:135–8. 10.1053/j.ajkd.2013.02.372 23602193

[75] Afrifa J Ogbordjor WD Duku-Takyi R. Variation in thyroid hormone levels is associated with elevated blood mercury levels among artisanal small-scale miners in Ghana. PLoS ONE. (2018) 13:e0203335. 10.1371/journal.pone.0203335 30161259 PMC6117084

[76] Chen A Kim SS Chung E Dietrich KN. Thyroid hormones in relation to lead, mercury, and cadmium exposure in the National Health and Nutrition Examination Survey, 2007–2008. Environ Health Perspect. (2012) 121:181–6. 10.1289/ehp.1205239 23164649 PMC3569681

[77] Kawada J Nishida M Yoshimura Y Mitani K. Effects of organic and inorganic mercurials on thyroidal functions. J Pharmacobio-Dyna. (1980) 3:149–59. 10.1248/bpb1978.3.149 6451681

[78] Iavicoli I Fontana L Bergamaschi A. The effects of metals as endocrine disruptors. J Toxicol Environ Health Part B. (2009) 12:206–23. 10.1080/10937400902902062 19466673

[79] Wadaan MA Effects of mercury exposure on blood chemistry and liver histopathology of male rats. Pharmacol Toxicol. (2009) 4:126–31. 10.3923/jpt.2009.126.131

[80] Chopra IJ. A study of extrathyroidal conversion of thyroxine (T4) to 3, 3′, 5-triiodothyronine (T3) *in vitro*. Endocrinology. (1977) 101:453–63. 10.1210/endo-101-2-453 18337

[81] Visser T Does-Tobé I Docter R Hennemann G. Subcellular localization of a rat liver enzyme converting thyroxine into tri-iodothyronine and possible involvement of essential thiol groups. Biochem J. (1976) 157:479–82. 10.1042/bj1570479 9074 PMC1163875

[82] Yoshida K Sugihira N Suzuki M Sakurada T Saito S Yoshinaga K. Effect of cadmium on T4 outer ring monodeiodination by rat liver. Environ Res. (1987) 42:400–5. 10.1016/S0013-9351(87)80206-2 3569180

[83] Brandão R Borges LP Nogueira CW. Concomitant administration of sodium 2, 3-dimercapto-1-propanesulphonate (DMPS) and diphenyl diselenide reduces effectiveness of DMPS in restoring damage induced by mercuric chloride in mice. Food Chem Toxicol. (2009) 47:1771–8. 10.1016/j.fct.2009.04.035 19406194

[84] Pyszel A Wrobel T Szuba A Andrzejak R. Effect of metals, benzene, pesticides and ethylene oxide on the haematopoietic system. Medycyna Pracy. (2005) 56:249–55. 16218139

[85] Lund B-O Miller DM Woods JS Studies on Hg (II)-induced H 2 O 2 formation and oxidative stress *in vivo* and *in vitro* in rat kidney mitochondria. Biochem Pharmacol. (1993) 45:2017–24. 10.1016/0006-2952(93)90012-L 8512585

[86] Peraza MA Ayala-Fierro F Barber DS Casarez E Rael LT. Effects of micronutrients on metal toxicity. Environ Health Perspect. (1998) 106:203. 10.1289/ehp.98106s1203 9539014 PMC1533267

[87] Naganuma A Furuchi T Miura N Hwang G-W Kuge S. Investigation of intracellular factors involved in methylmercury toxicity. Tohoku J Experi Med. (2002) 196:65–70. 10.1620/tjem.196.65 12498317

[88] Wang L Jia G. [Progress in developmental toxicity of methylmercury]. Wei sheng yan jiu = J Hygi Res. (2005) 34:633–5. 16329617

[89] Chen C Qu L Li B Xing L Jia G Wang T. Increased oxidative DNA damage, as assessed by urinary 8-hydroxy-2′-deoxyguanosine concentrations, and serum redox status in persons exposed to mercury. Clin Chem. (2005) 51:759–67. 10.1373/clinchem.2004.042093 15695327

[90] Maqbool F Niaz K Hassan FI Khan F Abdollahi M Health PC. Immunotoxicity of mercury: pathological and toxicological effects. J Environ Sci. (2017) 35:29–46. 10.1080/10590501.2016.1278299 28055311

[91] Silva IA Nyland JF Gorman A Perisse A Ventura AM Santos EC. Mercury exposure, malaria, and serum antinuclear/antinucleolar antibodies in Amazon populations in Brazil: a cross-sectional study. Environ Health. (2004) 3:11. 10.1186/1476-069X-3-11 15522122 PMC538267

[92] Suvd D Davaadorj R Baatartsol D Unursaikhan S Tsengelmaa M Oyu T Toxicity assessment in artisanal miners from low-level mercury exposure in Bornuur and Jargalant soums of Mongolia. Proc Environ Sci. (2015) 30:97–102. 10.1016/j.proenv.2015.10.017

[93] Tomicic C Vernez D Belem T Berode M. Human mercury exposure associated with small-scale gold mining in Burkina Faso. Int Arch Occupat Environ Health. (2011) 84:539–46. 10.1007/s00420-011-0615-x 21279378

